# Physical Properties, Antimicrobial Activity and In Vivo Tissue Response to Apexit Plus

**DOI:** 10.3390/ma13051171

**Published:** 2020-03-05

**Authors:** Roberto Alameda Hoshino, Guilherme Ferreira da Silva, Mateus Machado Delfino, Juliane Maria Guerreiro-Tanomaru, Mario Tanomaru-Filho, Estela Sasso-Cerri, Idomeo Bonetti Filho, Paulo Sérgio Cerri

**Affiliations:** 1 Department of Restorative Dentistry, Dental School—São Paulo State University (UNESP), Araraquara 14801-903, Brazil; robertohoshino@hotmail.com.br (R.A.H.); gferreiras@hotmail.com (G.F.d.S.); mateusdelfino@hotmail.com (M.M.D.); guerreiro.tanomaru@unesp.br (J.M.G.-T.); idomeo.bonetti@unesp.br (I.B.F.); 2 Laboratory of Histology and Embryology, Dental School—São Paulo State University (UNESP), Araraquara 14801-903, Brazil; estela.sasso@unesp.br

**Keywords:** biocompatibility, bioactivity, immunohistochemistry, alkaline phosphatase, *Enterococcus faecalis*, sealers

## Abstract

We investigated the physical properties, antimicrobial activity, and tissue reaction to Apexit Plus in comparison to Sealapex. Flow, radiopacity, setting time, and solubility were evaluated in each material. The antimicrobial activity against *Enterococcus faecalis* was performed. Polyethylene tubes containing Apexit Plus or Sealapex, and without material (control group) were implanted into the subcutaneous tissue of rats. At 7, 15, 30, and 60 days of implantation, the specimens were paraffin-embedded and the number of inflammatory cells (ICs) and the amount of birefringent collagen (BC) were quantified. The von Kossa reaction followed by immunohistochemistry for detection of alkaline phosphatase (ALP) was also performed. Statistical analysis was performed with ANOVA and Tukey test (*p* ≤ 0.05). The flow value of Apexit Plus was greater than Sealapex, whereas the radiopacity (3.44 mm Al) was lower than Sealapex (6.82 mm Al). Apexit Plus showed lower solubility and shorter initial and final setting (*p* < 0.0001), whereas the antimicrobial activity was significantly greater than Sealapex. Although the number of ICs was higher in Apexit Plus (*p* = 0.0009) at 7 days, no significant difference was detected between Apexit Plus and Sealapex at 15, 30, and 60 days. All groups showed higher values for BC in the capsules over time. ALP-immunolabelled cells were observed, mainly around von Kossa-positive structures, either in the capsules of Apexit Plus or Sealapex. Therefore, our results revealed that Apexit Plus exhibited a greater effectiveness against *Enterococcus faecalis* and better physical properties than Sealapex, except for the radiopacity. In vivo findings indicate that Apexit Plus is biocompatible and presents potential bioactivity in the subcutaneous tissue.

## 1. Introduction

The use of endodontic sealers with antibacterial activity has been effective in the clearance of persistent microorganisms [1] favoring periapical tissue repair [2]. Calcium hydroxide releases hydroxyl ion, providing an alkaline pH and exerting an antibacterial effect followed by periapical tissue repair [3,4]. Among several calcium hydroxide-based root canal filling materials, Sealapex (Sybron Endo, Glendora, CA, USA) is considered biocompatible [5]. This sealer contains calcium hydroxide in a polymeric matrix [1]. The base paste of this endodontic sealer contains calcium oxide, bismuth trioxide, zinc oxide, sub-micron silica, zinc stearate, titanium dioxide, and tricalcium phosphate. Its catalyst paste is constituted by blend, ethyl toluene sulfonamide, poly (methylene methyl salicylate) resin, and isobutyl salicylate [3]. Sealapex stimulates the deposition of calcified structure, inducing apical sealing after root canal treatment [5]. However, due to a prolonged setting time, this sealer presents high solubility [6], leading to non-homogeneous setting reaction forming a fragile matrix [7,8].

Apexit Plus (Ivoclar Vivadent, Schaan, Liechtenstein), a calcium hydroxide-based endodontic cement, has been launched in an attempt to provide a flawless seal at the apical foramen without damaging periodontal tissues. The base paste of Apexit Plus contains calcium hydroxide, calcium oxide, hydrated collophonium, highly dispersed silicon dioxide, and alkyl ester of phosphoric acid, while the activator paste contains disalicylate, bismuth hydroxide, bismuth carbonate associated to silicon dioxide, and phosphoric acid alkyl ester [4,9]. This calcium hydroxide-based sealer has caused slight cytotoxicity when cultured with L929 fibroblasts [10] and exerted good antimicrobial activity [11]. It has been suggested that the high pH provided by this sealer (to above 12.5) may be responsible for its antimicrobial effect [11,12]. Root canal filling materials should present some favorable psychochemical properties [6,7,8], including sufficient radiopacity to be distinguished from bone and teeth and an adequate flow capacity to be inserted into the root canal system [6]. Furthermore, cements may not present long setting time and high solubility, which reduce their resistance [7,8].

However, until now, no studies have been found showing the tissue response to Apexit Plus. Considering that cellular damage induced by endodontic sealers influences the outcome of endodontic therapy, the biocompatibility evaluation contributes to a better understanding of tissue reaction induced by endodontic materials. It has been demonstrated that polyethylene tubes filled with biomaterials implanted into subcutaneous tissues is an adequate methodology to investigate the biocompatibility of dental materials [13].

Besides biocompatibility, an endodontic sealer may also induce the formation of mineralized tissue formation. The von Kossa histochemical reaction has been widely used to detect the formation of calcium-containing structures in response to endodontic materials in the subcutaneous tissue, as an indicator of bioactivity [14,15,16]. A marker of mineralized tissue-producing cells, alkaline phosphatase (ALP), is an enzyme that catalyzes the hydrolysis of phosphomonoesters [17]. Considering that catalytic activity of ALP results in the increase of local concentration of inorganic phosphate (a mineralization promoter), this enzyme has been reported as a marker for osteogenic activity [18].

In the present study, we evaluated the flow, radiopacity, setting time, and solubility of Apexit Plus and Sealapex. The antimicrobial activity against *Enterococcus faecalis* and the subcutaneous tissue response induced by Apexit Plus in comparison to Sealapex were also evaluated. Consequently, the numerical density of ICs was estimated and the von Kossa reaction for the identification of calcium was carried out. In an attempt to evaluate whether these sealers are able to stimulate the mineralized tissue formation, the colocalization of von Kossa-positive structures and the osteoblast marker, ALP, was also investigated. The null hypothesis was that these root canal sealers would not present differences, either in the physical properties or in the biological response of the subcutaneous tissue.

## 2. Materials and Methods

### 2.1. Physical Analyses

#### 2.1.1. Flow

The flow analysis was conducted in accordance with ISO 6876/2012 [19]. After mixing, a 0.05 mL portion of each sealer (*n* = 6) was placed on a glass plate. After 180 min, a glass plate weighing 20 g was placed on the sealer and a load of 100 g was added on this plate, totaling a mass of 120 g, which was maintained on the sealer for 7 min. After load removal, with the help of a digital caliper the diameters of the material were measured. When the diameter discs were not uniformly circular and/or the difference between major and minor diameters was higher than 1 mm, the test was performed again. The test was performed three times for each material, and the mean value was calculated.

#### 2.1.2. Radiopacity

The radiopacity evaluation was performed according to ANSI/ADA Specification 57, standard for dental root-sealing materials [20]. For each material, 6 specimens with 10 mm diameter and 1.0 mm thickness were made and maintained at 37 °C for 48 h. The specimens were placed on occlusal radiographic films (Insight-Kodak Comp, Rochester, NY, USA) and exposed. An aluminum step wedge with variable thickness (from 2 to 16 mm, in 2 mm increments) was also placed on the radiographic films. Using an X-ray device (General Electric, Milwaukee, WI, USA) at 50 kvp, 10 mA, 18 pulses/s, and focus-film distance of 33.5 cm, the radiographic images were obtained. The radiographic films were revealed under standardized conditions and, subsequently, the images were digitalized. The radiographic analysis was performed with the help of an image program (ImageTool 3.0, UTHSCSA, San Antonio, TX, USA), using the methodology previously described [21].

#### 2.1.3. Setting Time

The setting time was obtained from 6 specimens per each material (10 mm diameter × 1 mm height) following regulations #57 of the American Dental Association (ADA) [20] and C266-03 of American Society for Testing and Materials (ASTM) [22]. After 150 ± 10 s to the onset of mixture, the initial setting time was obtained using a Gilmore needle of 100 ± 0.5 g and an active tip of 2.0 ± 0.1 mm diameter. The needle was positioned vertically on the material surface and when the needle did not mark on the material surface, it was considered as the initial setting time of the sealer. The final setting time was obtained using a Gilmore needle of 456 ± 0.5 g and an active tip of 1.0 ± 0.1 mm diameter as previously described [16,23]. The data were determined by the arithmetic mean of 6-repetition tests for each endodontic sealer.

### 2.2. Solubility

The solubility test was conducted according to ISO 6876/2012 [19]. The sealers were mixed and placed into molds of 1.5 mm thickness and 7.75 mm diameter, and a nylon thread was inserted in each specimen. Using a precision scale (BL 210S, Sartorius AG, Goettingen, Germany), the initial mass of each specimen was obtained. Each sealer sample was kept for 7 days suspended by nylon thread in a flask containing 10 mL deionized water at 37 °C. The sample was measured every 24 h until the stabilization of the mass values (constant weight). On the 7th day, after washing in distilled water the samples were maintained in a dehumidifier for 24 h. Then, the final mass of each sample was obtained; the loss of mass was calculated and expressed according to the percentage of the initial mass.

### 2.3. Biological Properties

#### 2.3.1. Antimicrobial Activity

*Enterococcus faecalis* (ATCC 29212) was used to investigate the antimicrobial activity by direct contact assay [24]. The *E. faecalis* was suspended in 4 mL brain heart infusion (BHI) medium for 6 h at 37 °C. Using a spectrophotometer (Model 600 Plus; Femto, São Paulo, SP, Brazil), the optical density of the bacterial suspension was adjusted to an equivalent concentration to 1.0 × 10^7^ CFU mL^−1^ (colony-forming units/mL). After mixing, 30 µL of Apexit Plus and Sealapex sealers were placed vertically in a 96-well microtiter plate. The sealer was placed in a standardized area on a side wall of the wells. The plate containing the sealers was maintained for 30 min in the laminar flux chamber under ultraviolet light and 10 µL of *Enterococcus faecalis* suspension was added in each well. As positive control, 10 µL of bacterial suspension were placed in the wells (without sealers). The plates were incubated for 1 h at 37 °C and, then, 200 µL BHI medium were added in each well. Colonies of surviving *E. faecalis* were calculated after serial dilution and plating for quantification of UFC/mL. Proceedings were performed in triplicate and with 6 parallels for each sealer evaluated. Data were transformed to log expression, and the mean and standard deviation was calculated for each group.

#### 2.3.2. Animal Experimental Procedures

The experimental protocol was submitted and approved by the Ethical Committee for Animal Research of Araraquara Dental School (FOAr-UNESP) following the Brazilian national law on animal use.

A total of 60 adult male Holtzman rats (*Rattus norvegicus albinus*) were randomly assigned into three groups of 20 animals each: Apexit Plus (Ivoclar Vivadent AG, Schaan, Liechtenstein), Sealapex (Sybron Endo, Glendora, CA, USA), and control group (empty polyethylene tubes). Polyethylene tubes (Embramed Ltda., São Paulo, SP, Brazil) measuring 10.0 mm length and 1.6 mm diameter were previously sterilized with ethylene oxide. The materials were mixed according to manufacturer’s instructions before their insertion into the polyethylene tubes. Thus, the tubes filled with Apexit Plus (*n* = 20) or Sealapex (*n* = 20) were implanted into dorsal subcutaneous tissue.

The rats received an intraperitoneal injection of ketamine hydrochloride (80 mg/kg of body weight) and xylazine hydrochloride (8 mg/kg of body weight). After the anesthesia, the dorsal skin was shaved and disinfected, and an incision measuring 1 cm was performed. In each subcutaneous pocket was placed one polyethylene tube. The rats were euthanized at 7, 15, 30, and 60 days after the tube implantation by an overdose of ketamine hydrochloride (240 mg/kg of body weight) and xylazine hydrochloride (24 mg/kg of body weight) solution. The implants surrounded by tissues were removed (*n* = 5/group/period) and fixed for 48 h in 4% formaldehyde (prepared from paraformaldehyde) buffered with 0.1 M sodium phosphate (pH 7.2).

After fixation, the specimens were gradually dehydrated with graded ethanol concentrations, cleared in xylene, and paraffin-embedded. From each specimen, 60 longitudinal sections (6 µm thick) were adhered to glass slides. Five slides containing non-serial sections, with a distance of at least 100 µm between the sections, were selected and stained with hematoxylin and eosin (H&E) for histological and quantitative analyses. Sections were submitted to the Picro Sirius red, von Kossa histochemical reaction, and von Kossa method followed by immunohistochemistry for the identification of alkaline phosphatase (ALP).

#### 2.3.3. Histological Analysis and Numerical Density of Inflammatory Cells

From H&E-stained sections, the histological description and intensity of inflammatory reaction in the capsules were carried out. These analyses were performed in all rats (*n* = 5 per group/period). The inflammatory cells was counted in the standardized field (0.09 mm^2^) obtained from the central portion of the capsule, at ×695 magnification. The number of ICs (neutrophils, lymphocytes, macrophages, and plasma cells) was obtained with the help of an image analysis program (Image Pro-Express 6.0, Olympus, Tokyo, Japan). For each specimen, the numerical density of ICs was estimated from three non-serial sections (total area of 0.27 mm^2^) [16,25].

#### 2.3.4. Quantification of Collagen Fibers

The amount of collagen was quantified in the sections submitted to 0.1% Picro Sirius red and analyzed under polarized light microscope. From each specimen, two sections with minimal distance of at least 100 µm were selected. In each section, a birefringent image of the capsule was obtained at ×695 magnification (standardized field about 0.09 mm^2^). All the birefringent images were obtained under the same standardized conditions, such as light intensity, diaphragm aperture, condenser position, and exposition time. The birefringent content in the capsules was calculated using ImageJ^®^ (National Institutes of Health, Bethesda, MD, USA) [26].

#### 2.3.5. Von Kossa Histochemical Reaction

Detection of calcium salts was detected by the von Kossa method, as previously described [15,16,25]. Two non-serial sections per specimen were subjected to the von Kossa method and were subsequently stained with Picro Sirius red and analyzed using a light microscope. In addition, unstained sections were also analyzed under an Olympus microscope with polarization filters to evaluate the presence of birefringent structures in the capsules [27].

#### 2.3.6. Von Kossa Method Combined with Immunohistochemical Detection of Alkaline Phosphatase

Deparaffinized sections were first subjected to the von Kossa method followed by immunohistochemistry for the detection of ALP. For antigen retrieval, the sections were immersed for 20 min in sodium citrate buffer pH 6.0 at 96–98 °C. After a cooling-off period, the endogenous peroxidase was blocked with 5% hydrogen peroxide for 20 min. The sections were washed and treated with 2% bovine serum albumin (Sigma-Aldrich Chemie) for 20 min. After incubation at 4 °C for 16–18 h with rabbit anti-alkaline phosphatase antibody (Santa Cruz Biotechnology, Santa Cruz, CA, USA) diluted at 1:400, the sections were incubated with Super Sensitive^TM^ IHC Detection Systems (BioGenex Laboratories, Fremont, CA, USA). The immunoreaction was revealed by 3,3′-diaminobenzidine (DAB, Biocare Medical Inc., Concord, CA, USA) for 3 min and, subsequently, the sections were counterstained with hematoxylin. A non-immune serum, instead of primary antibody, was used as negative control.

### 2.4. Statistical Analysis

Data of flow, radiopacity, setting time, solubility, and antimicrobial activity were subjected to the one-way ANOVA for the analysis of variance followed by Tukey’s post-hoc test. Numerical density of ICs and the amount of collagen were subjected to the D’Agostino–Pearson omnibus normality test, which detected normal Gaussian distribution of the data. Subsequently, these data were subjected to the two-way ANOVA analysis and Tukey’s post-hoc test. The significance level accepted was *p* ≤ 0.05. Statically analysis was performed using GraphPad Prism 6.01 software (GraphPad Software, Inc., La Jolla, CA, USA).

## 3. Results

### 3.1. Physical Analyses 

#### 3.1.1. Flow and Radiopacity Analyses

As shown in Table 1, Apexit Plus and Sealapex showed values according to ISO 6876/2012 [19], which attests that sealer discs must have a flow diameter of at least 17 mm. However, Apexit Plus had a significantly greater flow value than Sealapex (*p* < 0.0001).

Although both sealers had radiopacity values above the minimum value (3 mm Al) set by ANSI/ADA Specification 57 [20], the radiopacity of Apexit Plus (3.44 mm Al) was significantly lower (*p* < 0.0001) than the Sealapex (6.82 mm Al), as shown in Table 1.

#### 3.1.2. Setting Time and Solubility

According to Table 2, Apexit Plus showed significantly reduced values of setting time when compared with Sealapex (*p* < 0.0001). The solubility of Apexit Plus was significantly decreased in comparison with Sealapex (*p* < 0.0001).

### 3.2. Biological Properties

#### 3.2.1. Antimicrobial Activity

**Figure 1:**
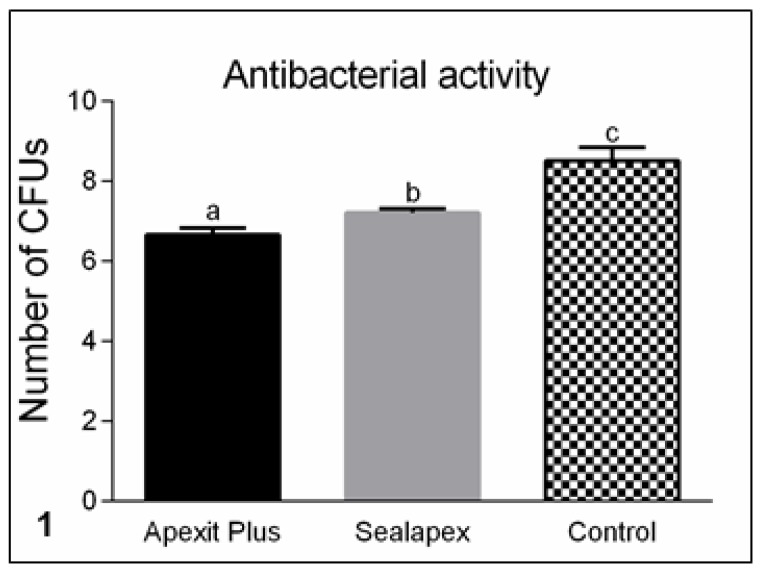
Number of colony-forming units (CFUs) of *Enterococcus faecalis* (ATCC 29212) after direct contact with freshly mixed sealers (Apexit Plus and Sealapex) and control samples. The CFUs are shown in logarithmic graduation. Different superscript letters (a, b, and c) indicate significant differences among the groups (*p* < 0.05). Apexit Plus ≠ Sealapex; Apexit Plus ≠ Control; Sealapex ≠ Control.

According to Figure 1, the direct contact test of *E. faecalis* with Apexit Plus and Sealapex caused a statistical reduction of colony-forming units (CFUs). Moreover, the antimicrobial activity of Apexit Plus was significantly greater than that of Sealapex (*p* < 0.05).

#### 3.2.2. Morphological Analysis and Numerical Density of Inflammatory Cells

**Figure 2:**
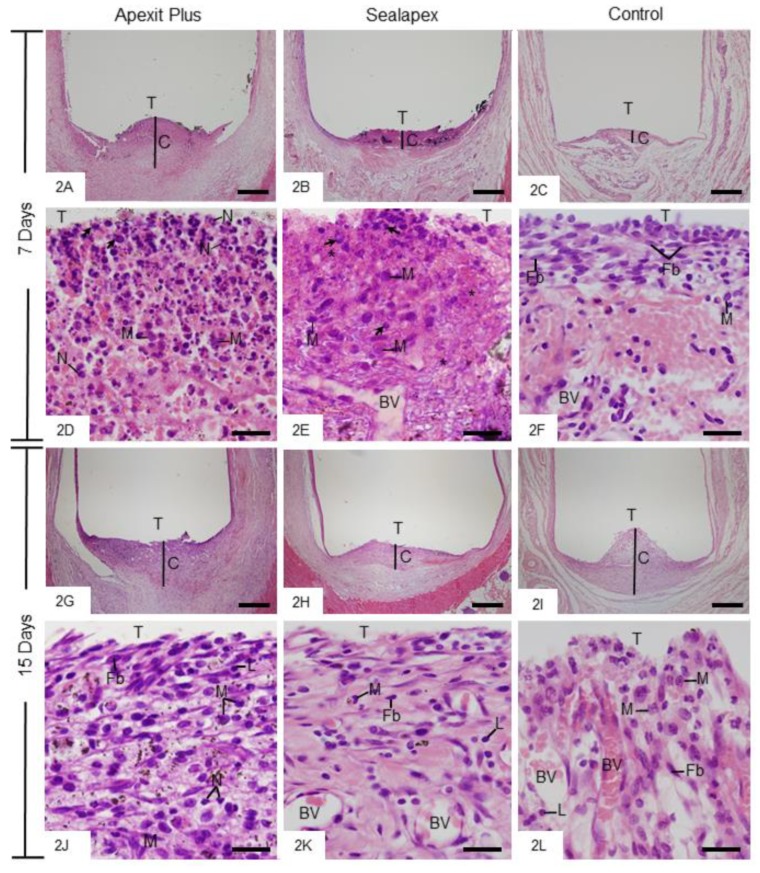
Photomicrographs show portions of capsules (C) surrounding the opening of the tubes (T) at 7 days. 2**A**–**C** General views of capsules with evident inflammatory infiltrate, mainly in the Apexit Plus (Figure 2A) and Sealapex (Figure 2B) groups. Bars: 500 µm. 2**D**–**F** High magnifications of the innermost portion of capsules. Several neutrophils (N) and macrophages (M) are seen in Apexit Plus (Figure 2D) and Sealapex (Figure 2E) in comparison with the control group (Figure 2F). Cells exhibiting condensed chromatin (arrows) and areas with remnants of degenerate material (asterisks) are also observed. BV, blood vessels; Fb, fibroblast. Bars: 20 µm. 2**G**–**L** Light micrographs exhibit portions of capsules at 15 days. 2**G**–**I** General view of well-defined capsules (C). Bars: 500 µm. At high magnification (Figure 2J–L), the capsules contain fibroblasts (Fb) among macrophages (M), lymphocytes (L), and several blood vessel profiles (BV). N, neutrophils. H&E. Bars: 20 µm.

**Figure 3:**
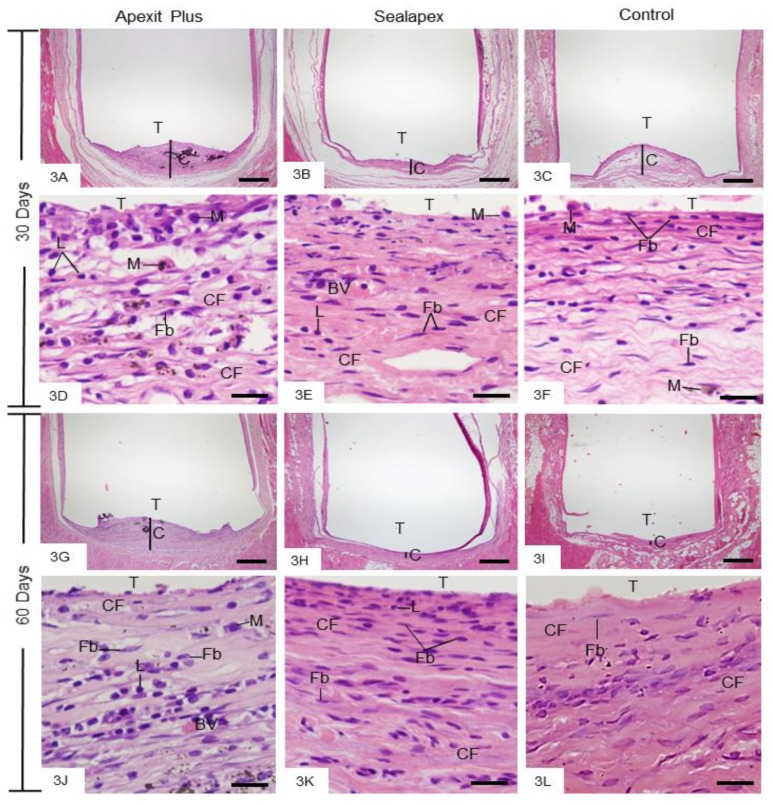
Photomicrographs show portions of capsules surrounding the opening of the tubes (T) at 30 (3**A**–**F**) and 60 (3**G**–**L**) days. Low magnifications (3**A**–**C** and 3**G**–**I**) show thick capsules (C) in Apexit Plus (3**A**,**G**) in comparison with Sealapex (3**B**,**H**) and control (3**C**,**I**) groups. 3**D**–**F** (30 days) and 3**J**–**L** (60 days) High magnifications. 3**D**–**F** Macrophages (M), lymphocytes (L), fibroblasts (Fb), and collagen fibers (CF) are present. 3**J**–**L** The capsules contain numerous fibroblasts (Fb) and bundles of collagen fibers (CF). M, macrophages; L, lymphocytes; BV, blood vessel profile. H&E. Bars: 500 µm (3**A**–**C** and 3**H**–**J**) and 20 µm (3**D**–**F** and 3**J**–**L**).

After 7 days, the capsules showed well-defined structure with variable thickness. However, the capsules of the Apexit Plus and Sealapex groups were thicker than the control group (Figure 2A–C). The inflammatory reaction in the capsules of Apexit Plus contained several neutrophils (Figure 2D), whereas in Sealapex they contained mainly macrophages (Figure 2E). The capsules exhibited areas with remnants of degenerate material and some cells with pyknotic nucleus (Figure 2D,E). In the control group, the capsules exhibited some macrophages among fibroblasts and an intense vascular network (Figure 2F). After 15 days of implantation, the capsules of Apexit Plus (Figure 2G) were thicker than those of Sealapex Plus (Figure 2H); in the control group, the connective tissue was often invaginated into the tubes (Figure 2I). Several macrophages and lymphocytes were still observed in Apexit Plus (Figure 2J), whereas in the Sealapex (Figure 2K) and control (Figure 2L) specimens, the capsules exhibited fibroblasts and several blood vessels. At 30 and 60 days, the capsules surrounding Apexit Plus (Figure 3A,G) were still thick when compared with the Sealapex (Figure 3B,H) and control (Figure 3C,I) groups. At 30 days, the Apexit Plus specimens exhibited several macrophages and lymphocytes among few fibroblasts and collagen fibers (Figure 3D). In contrast, numerous fibroblasts among collagen bundles were present in Sealapex (Figure 3E) and control (Figure 3F) groups. At 60 days, the capsules surrounding the sealers showed few inflammatory cells among numerous fibroblasts and bundles of collagen fibers (Figure 3J,K). In the control group, the mild inflammatory infiltrate was almost unnoticed in the fibrous capsules (Figure 3L).

The quantitative analysis (Table 3) showed that the highest values of ICs were verified in the capsules of all groups at 7 days. Although the number of ICs was greater in the Apexit Plus than in the Sealapex specimens at 7 days (*p* = 0.0009), no significant difference was detected between these groups at 15 (*p* = 0.189), 30 (*p* = 0.087), and 60 days (*p* = 0.087). At all periods, the number of ICs was significantly greater in Apexit Plus (*p* < 0.0001) and Sealapex (*p* ≤ 0.0449) than in the control group.

#### 3.2.3. Birefringent Collagen

**Figure 4:**
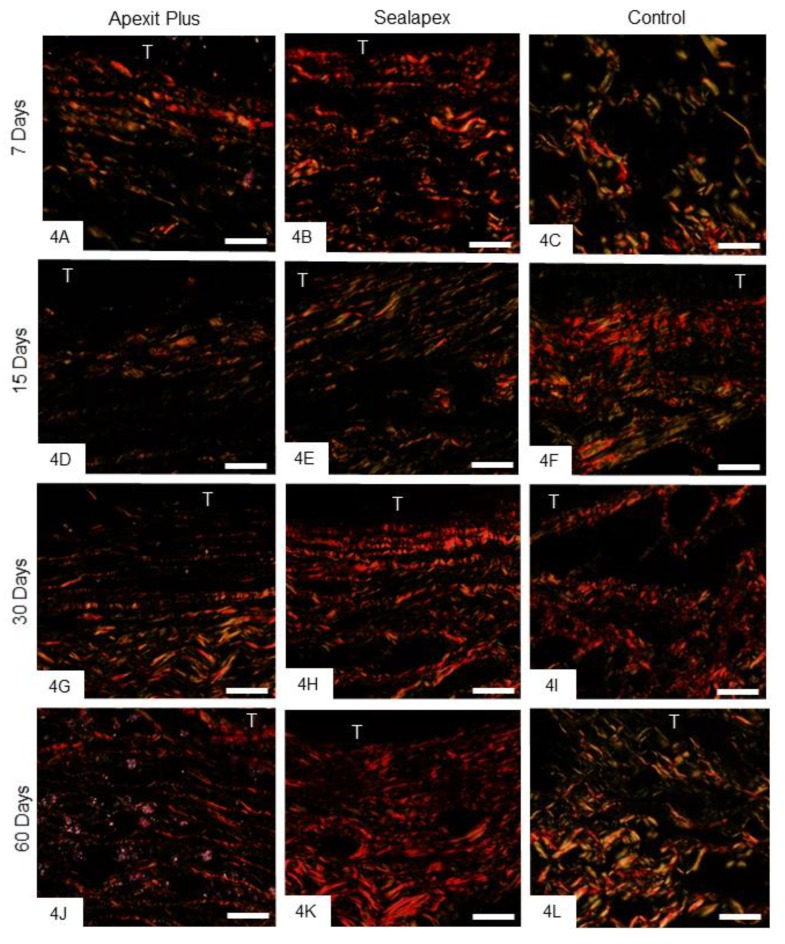
Photomicrographs show sections subjected to the Picro Sirius red and analyzed under polarized light. At 7 (4**A**–**C**) and 15 (4**D**–**F**) days, the capsules contain little birefringent material (orange, red, yellow). At 30 (4**G**–**I**) and 60 (4**J**–**L**) days, an evident increase in the amount of birefringent material is seen in the capsules. T, tube opening. Bars: 25 µm.

At 7 and 15 days, there was little birefringent material in the capsules (Figure 4A–F), whereas an evident increase in this material was seen at 30 (Figure 4G–I) and 60 (Figure 4J–L) days, in all groups. The quantitative findings (Table 3) showed that the amount of birefringent material increased significantly (*p* < 0.0006) in all the specimens over time. At all periods, the amount of birefringent collagen was significantly greater around the Sealapex specimens than Apexit Plus (*p* < 0.0005), whereas no significant difference was detected between Sealapex and the control group (*p* = 0.310).

#### 3.2.4. Von Kossa Histochemical Method and Analysis under Polarized Light Microscope

**Figure 5:**
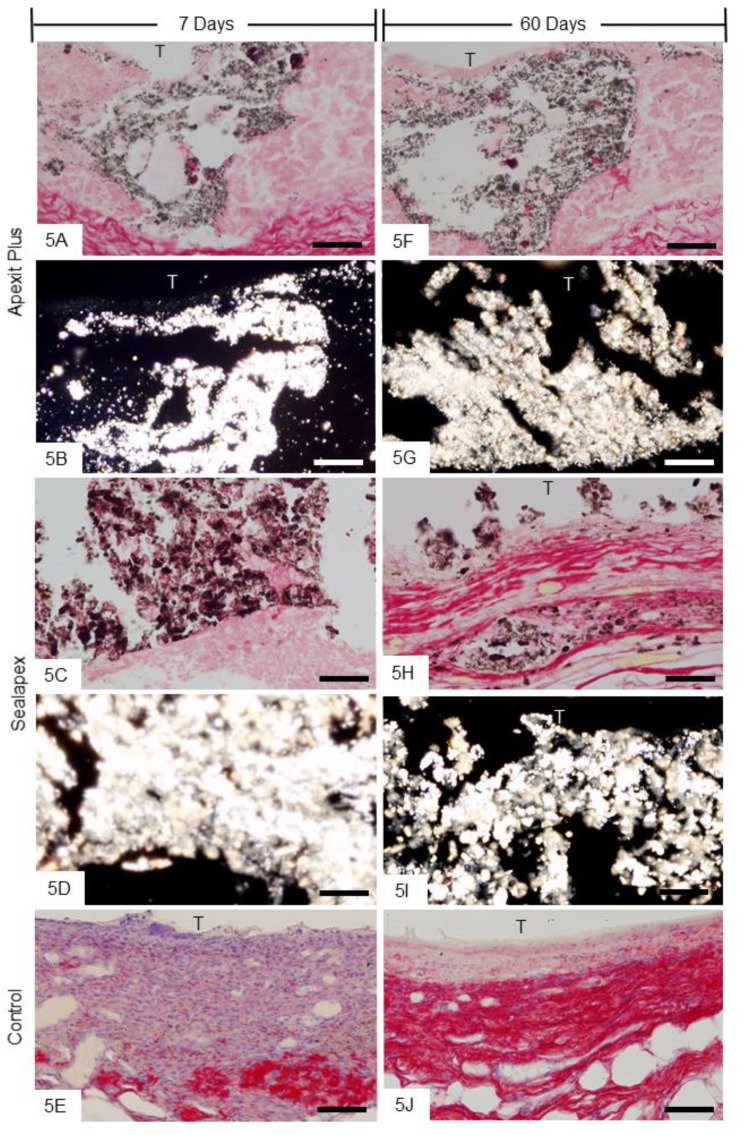
Photomicrographs show capsules next to the opening of the tubes. **A**,**C**,**E**,**F**,**H**,**J** The von Kossa histochemical method and Picro Sirius red; von Kossa-positive structures (black) are present in Apexit Plus (**A**,**F**) and Sealapex (**C**,**H**) groups. Note that no von Kossa-positive structure is observed in control specimens (**E**,**J**). **B**,**D**,**G**,**I** Light micrographs showing unstained sections analyzed under polarized light. The capsules of Apexit Plus (**B**,**G**) and Sealapex (**D**,**I**) groups exhibit birefringent structures. T, space of the implanted tube. Bars: 25 µm.

Sections of capsules of Apexit Plus (Figure 5A,F) and Sealapex (Figure 5C,H) exhibited von Kossa-positive structures (black). Sections unstained, close to those subjected to the von Kossa method, analyzed under polarized light exhibited birefringent structures in similar regions of the capsules from Apexit Plus (Figure 5B,G) and Sealapex (Figure 5D,I). Furthermore, von Kossa-positive structures were not observed in the control specimens (Figure 5E,J).

#### 3.2.5. Von Kossa Method Combined with Immunohistochemical Detection of ALP

**Figure 6:**
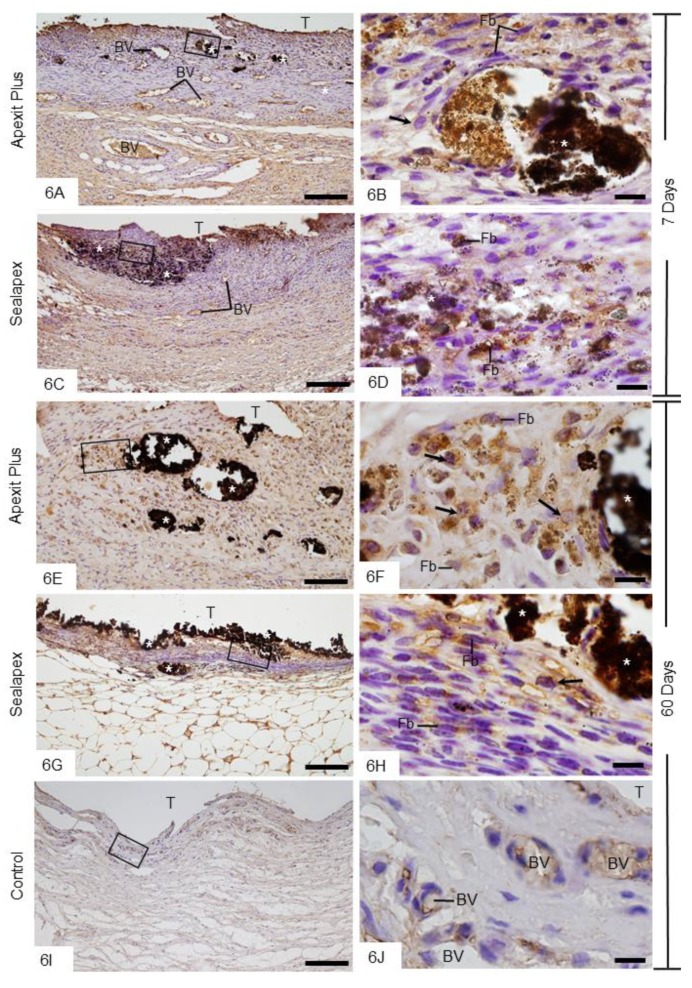
Photomicrographs show sections submitted to the von Kossa method (black) followed by immunohistochemistry for detection of ALP (brown-yellow) and counterstained with hematoxylin. **A**,**C**,**E**,**G** General view of the capsules showing von Kossa-positive structures (asterisks). Note that ALP-immunolabelled cells are located mainly next to the von Kossa-positive structures (asterisks) and in the internal surface of capsules. **B**,**D**,**F**,**H** Higher magnifications showing outlined area of the general view. Fibroblasts (Fb) and round/ovoid cells (arrows) show strongly ALP-immunolabelled cytoplasm (brown-yellow). **I**,**J** None or weak ALP immunolabelling is present in the capsule of control group. In Figure 6J, outlined area of Figure 6I, some vascular cells exhibit subtle ALP immunoreactivity. T, space of the implanted tube; BV, blood vessel profiles. Bars: 150 µm (**A**,**C**,**E**,**G**,**I**) and 10 µm (**B**,**D**,**F**,**H**,**J**).

When the sections were subjected to the von Kossa method followed by ALP immunohistochemistry, an evident immunoreaction (brown-yellow) was seen mainly next to the von Kossa-positive structures as well as in the internal surface of the capsules, that is, in juxtaposition to the Apexit Plus (Figure 6A,E) and Sealapex (Figure 6C,G) sealers, at all time points. The extensive examination revealed fibroblasts and round/ovoid cells with ALP-immunolabelled cytoplasm in the capsules of Apexit Plus (Figure 6B,F) and Sealapex (Figure 6D,H). In the control group, no reaction product for the von Kossa method was observed, whereas a subtle ALP immunoreactivity was observed in the vascular cells (Figure 6I,J). ALP immunolabelling was not seen in the negative control sections (data not shown).

## 4. Discussion

After root canal preparation, it is important to completely fill the root canal system to ensure clinical success over time. The choice of endodontic sealers depends on their capacity to provide an adequate sealing and stimulate periodontium tissue formation. Thus, the ideal endodontic sealer should exhibit adequate physicochemical and biological properties and should be easy to manipulate. Apexit Plus was launched in an attempt to provide a flawless seal at the apical foramen as well as to allow periodontal tissue repair. In the present study, some physical properties of Apexit Plus were evaluated; we also evaluated whether this endodontic sealer is biocompatible and potentially bioactive.

### 4.1. Physical Properties

An adequate endodontic sealer must present radiopacity to be distinguished from bone and teeth and to facilitate the evaluation of the root filling’s quality [28]. Here, the radiopacity of Apexit Plus and Sealapex was greater than 3 mm of aluminum as recommended by ANSI/ADA Specification 57 [20]. In its composition, Apexit Plus contains bismuth-rich particles as radiopacifier [29], resulting in a radiopacity medium value above 3.44 mmAl. Similar values were also found by other studies [6,29]. However, Sealapex containing lanthanum dioxide (20%) in addition to bismuth trioxide [30] exhibited a radiopacity value of about 6.82 mm Al. In fact, Sealapex has demonstrated greater radiopacity than Apexit Plus [6].

The setting time of the sealers evaluated according the ANSI/ADA Specification 57 [20] revealed that setting time of Apexit Plus was reduced in comparison with that of Sealapex. The greatest difference was observed at initial setting time, since the initial setting time of Apexit Plus occurred after around 5 h, whereas the initial setting time of Sealapex was only observed after 19 h. The final setting of Apexit Plus occurred almost around 18 h and, for Sealapex, it occurred after 21 h. Although there is no definitive standard for the setting time of endodontic sealers, it has been suggested that a prolonged setting time may be responsible for the increase solubility. In fact, Sealapex exhibited greater solubility than Apexit Plus, reinforcing the concept that the extended setting time leads to an increase in solubility. The delay in the setting time of Sealapex has been associated with calcium oxide in its composition; the calcium oxide in contact with water gives rise to calcium hydroxide, which delays the setting time process of the sealer [6]. Moreover, the high solubility of Sealapex has been attributed to the non-homogeneous setting reaction of this sealer, forming a fragile matrix [7,8] with low dimensional stability [31] due to high water absorption [32].

According to the ISO 6876/2012 [19], the endodontic sealers must exhibit the minimum value for flow of 17 mm. Our findings revealed that both sealers showed values for flow within the specification of the ISO 6876/2012 [19]. However, Apexit Plus had a higher flow value (around 25 mm) than that of Sealapex (around 20 mm). It is important to emphasize that a greater flow rate allows an adequate filling of the root canal; however, an excessive flow is not ideal, as it increases the risk of apical extrusion of the sealer [33].

### 4.2. Biological Properties

Our findings showed that Apexit Plus had an effective antibacterial action against *Enterococcus faecalis,* a facultative anaerobic Gram-positive coccus responsible for root canal treatment failures [34]. Here, the findings were obtained using the direct contact test for 60 minutes, a widely used methodology performed to assess the antimicrobial activity of materials [35,36]. The antimicrobial properties of calcium hydroxide-based sealers has been related to hydroxyl ion release and, consequently, to an increased alkaline pH in the environment promoted by fresh sealers [4]. Some studies have reported that, in the first hours, Apexit Plus provides a highly alkaline pH (about 12) to the medium [12]. Considering that Sealapex initially provides a pH around 7.18 to the medium [30], it is possible that the high effectiveness of Apexit Plus against *Enterococcus faecalis* may be due to the elevated pH initially provided by this endodontic sealer. However, the antimicrobial effectiveness of Apexit Plus may decrease over time, since the pH decreases when the hydroxide calcium-based sealers take hold, culminating in a significant loss of antimicrobial efficacy of these sealers.

Although Apexit Plus recruited a higher number of inflammatory cells when compared with Sealapex, inflammatory reaction decreased over time. It is conceivable to suggest that the high pH of Apexit Plus may induce a more accentuated inflammatory reaction when compared with Sealapex. Moreover, it is possible that irritant substances, such as disalicylate and alkyl ester of phosphoric acid, may be released by Apexit Plus, promoting an inflammatory reaction more accentuated than Sealapex over the short term (7 days). At 7 days, the capsules of Apexit Plus exhibited cells with pyknotic nucleus (i.e., condensed chromatin), indicating that this sealer may initially exert cell damage to the subcutaneous connective tissue. It is important to emphasize that, in the Sealapex specimens, acidophilic areas (typical of tissue degeneration) were also observed. Macrophages were also found in these areas of capsules, indicating that the cellular debris and remnants of extracellular matrix may be internalized by phagocytic cells. However, a reduction in the numerical density of the inflammatory infiltrate was verified in the capsules over time, suggesting that the damage promoted by Apexit Plus is transitional. In fact, differences in the inflammatory infiltrate were not seen between Apexit Plus and Sealapex at 15, 30, and 60 days. From 7 to 15 days, the inflammatory infiltrate reduced significantly in Apexit Plus, whereas in the Sealapex specimens this reduction was not significant. It is possible that the maintenance of the inflammatory reaction in the Sealapex specimens at 15 days occurred due to its high solubility [8]. The cytotoxicity of Sealapex freshly prepared on MG-63 cells and human gingival fibroblasts is maintained for a longer period than AH Plus and EndoSeal [37].

Apexit Plus shows low or no cytotoxicity effect on L929 mouse fibroblasts [10] and human periodontal ligament fibroblasts [9]. Moreover, no genotoxicity was induced by Apexit Plus on an immortalized cell line derived from human pulp [38]. Our morphological analysis showed that inflammatory cells were gradually replaced by fibroblasts distributed among bundles of collagen fibers in the capsules of all groups over time. This idea is supported since, from 7 to 60 days, the amount of birefringent collagen increased significantly in the Apexit Plus and Sealapex specimens, similarly to that observed in the control group. Thus, Apexit Plus and Sealapex allowed the replacement of the inflammatory reaction by connective tissue, indicating that these sealers are biocompatible [25,26,39].

Our histochemical findings showed von Kossa-positive structures in the Apexit Plus and Sealapex specimens indicating, therefore, the calcium precipitation in the capsules [15,16,25,27]. Furthermore, the examination under polarized light revealed birefringent structures in the capsules from unstained sections next to those submitted to the von Kossa method. It is well established that calcium ions bond with the carbon dioxide from tissue fluid, generating calcium carbonate crystals, which exhibit birefringence [27]. Calcium ions are essential for the formation of hydroxyapatite [40] and exert an important role in biomineralization [5]. Moreover, calcium ions are also essential for proliferation and differentiation of mineralized tissue-producing cells [41]. Here, strong ALP immunolabelling was predominantly observed in the cells close to the von Kossa-positive structures. Since ALP at alkaline pH releases inorganic phosphate [17,41], researchers have suggested a pivotal role played by this enzyme in the early stages of hard tissue formation. Considering that Apexit Plus and Sealapex release hydroxyl and provide an alkaline pH to the medium [11,31], it is not surprising that these sealers stimulate the ALP expression by cells of the subcutaneous connective tissue, as observed in the present study. This evidence is confirmed by the fact that immunostained cells were not seen in the connective tissue of the control group, except in vascular cells. Endothelial cells can, occasionally, exhibit ALP immunoexpression as reported in the literature [41,42]. In the control group, the ALP-immunolabelled cells may have a participation in the regression of the inflammatory infiltrate and tissue remodeling, since alkaline phosphatase secreted by endothelial cells acts as an anti-inflammatory agent stimulating wound healing [42]. In conclusion, our findings indicate that Apexit Plus is biocompatible, favors calcium precipitation, and may stimulate the osteogenic potential by subcutaneous connective tissue cells in rats.

## 5. Conclusions

In the present study, Apexit Plus exhibited adequate radiopacity, solubility, and flow values. Moreover, Apexit Plus showed a reduced initial setting time and greater antibacterial activity against *Enterococcus faecalis* than Sealapex. The analyses of the tissue response to Apexit Plus revealed that this sealer is biocompatible and exerts potential bioactivity.

## Figures and Tables

**Table 1:** Means and standard deviations of the flow test (mm) and radiopacity (mm Al) of the Apexit Plus and Sealapex sealers.

Sealers	Flow Test	Radiopacity
Apexit Plus	25.69 ± 1.12 ^a^	3.44 ± 0.32 ^a^
Sealapex	20.93 ± 1.12 ^b^	6.82 ± 0.68 ^b^

Different superscript letters (^a^ and ^b^) in the columns indicate significant differences between the sealers (*p* < 0.05). Flow test: Apexit Plus ≠ Sealapex. Radiopacity: Apexit Plus ≠ Sealapex.

**Table 2:** Means and standard deviations of initial and final setting time (min) and final solubility (%) of the Apexit Plus and Sealapex sealers.

Sealers	Setting Time	Solubility
	Initial Final	Final (%)
Apexit Plus	298 ± 13.51 ^a^ 1062 ± 17.62 ^a^	−1.333 ± 0.2364 ^a^
Sealapex	1181 ± 14.32 ^b^ 1268 ± 22.80 ^b^	−6.952 ± 1.302 ^b^

Different superscript letters (^a^ and ^b^) in the columns indicate significant differences between the sealers (*p* < 0.05). Initial setting time: Apexit Plus ≠ Sealapex. Final setting time: Apexit Plus ≠ Sealapex. Solubility: Apexit Plus ≠ Sealapex.

**Table 3:** Number of inflammatory cells (ICs) per mm^2^ and content (in percentage) of birefringent collagen (BC) in the capsules of the Apexit Plus, Sealapex, and control groups.

		Apexit Plus	Sealapex	Control
7 days	ICs BC	580 ± 145 ^a;1^ 27.4 ± 2.4 ^a;1^	425 ± 71 ^b;1^ 32.5 ± 1.8 ^b;1^	327 ± 45 ^c;1^ 30.0 ± 1.7 ^ab;1^
15 days	ICs BC	445 ± 35 ^a;2^ 28.6±1.4 ^a;1^	377 ± 38 ^a;1^ 35.7 ± 5.3 ^b;1^	181 ± 30 ^b;2^ 39.3 ± 3.0 ^b;2^
30 days	ICs BC	413 ± 30 ^a;2^ 32.0 ± 2.3 ^a;2^	322 ± 32 ^a;1^ 44.6 ± 5.5 ^b;2^	156 ± 30 ^b;2^ 42.6 ± 2.7 ^b;2^
60 days	ICs BC	300 ± 31 ^a;3^ 34.0 ± 1.2 ^a;2^	204 ± 21 ^a;2^ 49.1 ± 4.7 ^b;2^	93 ± 45 ^b;2^ 54.2 ± 3.8 ^b;3^

Mean (standard deviation). The comparison between groups in the same period is indicated by superscript letters in the line; same letters = no statistically significant difference. ^a^ ≠ ^b^ ≠ ^c^. The analysis of data of one group between the time points is indicated by superscript numbers in the column; same numbers = no statistically significant difference. ^1^ ≠ ^2^ ≠ ^3^. Tukey test (*p* ≤ 0.05).

## Notes

**Compliance with ethical standards**

## References

[1] Rezende G.C., Massunari L., Queiroz I.O., Gomes-Filho J.E., Jacinto R.C., Lodi C.S., Dezan-Junior E. (2016). Antimicrobial action of calcium hydroxide-based endodontic sealers after setting, against *E. faecalis* biofilm. Braz. Oral Res..

[2] Poggio C., Trovati F., Ceci M., Colombo M., Pietrocola G. (2017). Antibacterial activity of different root canal sealers against *Enterococcus faecalis*. J. Clin. Exp. Dent..

[3] Ballullaya S.V., Vinay V., Thumu J., Devalla S., Bollu I.P., Balla S. (2017). Stereomicroscopic dye Leakage measurement of six different root canal sealers. J. Clin. Diag. Res..

[4] Pawinska M., Szczurko G., Kierklo A., Sidun J. (2017). A laboratory study evaluating the pH of various modern root canal filling materials. Adv. Clin. Exp. Med..

[5] Bueno C.R., Valentim D., Marques V.A., Gomes-Filho J.E., Cintra L.T., Jacinto R.C., Dezan-Junior E. (2016). Biocompatibility and biomineralization assessment of bioceramic-, epoxy-, and calcium hydroxide-based sealers. Braz. Oral Res..

[6] Marín-Bauza G.A., Silva-Sousa Y.T., da Cunha S.A., Rached-Junior F.J., Bonetti-Filho I., Sousa-Neto M.D., Miranda C.E. (2012). Physicochemical properties of endodontic sealers of different bases. J. Appl. Oral Sci..

[7] Schafer E., Zandbiglari T. (2003). Solubility of root-canal sealers in water and artificial saliva. Int. Endod. J..

[8] Borges R.P., Sousa-Neto M.D., Versiani M.A., Rached-Junior F.A., De-Deus G., Miranda C.E., Pecora J.D. (2012). Changes in the surface of four calcium silicate-containing endodontic materials and an epoxy resin-based sealer after a solubility test. Int. Endod. J..

[9] Szczurko G., Pawinska M., Luczaj-Cepowicz E., Kierklo A., Marczuk-Kolada G., Holownia A. (2018). Effect of root canal sealers on human periodontal ligament fibroblast viability: Ex vivo study. Odontology.

[10] Konjhodzic-Prcic A., Jakupovic S., Hasic-Brankovic L., Vukovic A. (2015). Evaluation of biocompatibility of root canal sealers on L929 fibroblasts with Multiscan EX Spectrophotometer. Acta Inform. Med..

[11] Vanapatla A., Vemisetty H., Punna R., Veeramachineni C., Venkata R.P., Muppala J.N., Dandolu R. (2016). Comparative evaluation of antimicrobial effect of three endodontic sealers with and without antibiotics—An in vitro study. J. Clin. Diag. Res..

[12] Slutzky-Goldberg I., Slutzky H., Solomonov M., Moshonov M., Weiss E.I., Matalon S. (2008). Antibacterial properties of four endodontic sealers. J. Endod..

[13] International Organization for Standardization (2016). Biological Evaluation of Medical Devices. Part 6: Tests for Local Effects after Implantation.

[14] Gomes-Filho J.E., Bernabé P.F., Nery M.J. (2008). Reaction of rat connective tissue to a new calcium hydroxide-based sealer. Oral Surg. Oral Med. Oral Pathol. Oral Radiol. Endod..

[15] Viola N.V., Guerreiro-Tanomaru J.M., da Silva G.F., Sasso-Cerri E., Tanomaru-Filho M., Cerri P.S. (2012). Biocompatibility of an experimental MTA sealer implanted in the rat subcutaneous: Quantitative and immunohistochemical evaluation. J. Biomed. Mater. Res. B Appl. Biomater..

[16] Silva G.F., Bosso R., Ferino R.V., Tanomaru-Filho M., Bernardi M.I., Guerreiro-Tanomaru J.M., Cerri P.S. (2014). Microparticulated and nanoparticulated zirconium oxide added to calcium silicate cement: Evaluation of physicochemical and biological properties. J. Biomed. Mater. Res. A.

[17] Holtz K.M., Kantrowitz E.R. (1999). The mechanism of the alkaline phosphatase reaction: Insights from NMR, crystallography and site-specific mutagenesis. FEBS Letters..

[18] Golub E.E., Boesze-Battaglia K. (2007). The role of alkaline phosphatase in mineralization. Curr. Opi. Orthop..

[19] (2012). Dental Root Canal Sealing Materials.

[20] (2000). Laboratory Testing Methods: Endodontic Filling and Sealing Materials.

[21] Hungaro-Duarte M.A., de Oliveira E., Kadre G.D., Vivan R.R., Guerreiro-Tanomaru J.M., Tanomaru-Filho M., de Moraes I.G. (2009). Radiopacity of portland cement associated with different radiopacifying agents. J. Endod..

[22] (2000). Standard Test Method for Time and Setting of Hydraulic-Cement Paste.

[23] Mendes A.T., Silva P.B.D., Só B.B., Hashizume L.N., Vivan R.R., Rosa R.A.D., Duarte M.A.H., Só M.V.R. (2018). Evaluation of physicochemical properties of new calcium silicate-based sealer. Braz. Dent. J..

[24] Arias-Moliz M.T., Ruiz-Linares M., Cassar G., Ferrer-Lugue C.M., Baca P., Ordinola-Zapata R., Camilleri J. (2015). The effect of benzalkonium chloride additions to AH Plus sealer. Antimicrobial, physical and chemical properties. J. Dent..

[25] Silva G.F., Tanomaru-Filho M., Bernardi M.I., Guerreiro-Tanomaru J.M., Cerri P.S. (2015). Niobium pentoxide as radiopacifying agent of calcium silicate-based material: Evaluation of physicochemical and biological properties. Clin. Oral Investig..

[26] Saraiva J.A., da Fonseca T.S., da Silva G.F., Sasso-Cerri E., Guerreiro-Tanomaru J.M., Tanomaru-Filho M., Cerri P.S. (2018). Reduced interleukin-6 immunoexpression and birefringent collagen formation indicate that MTA Plus and MTA Fillapex are biocompatible. Biomed. Mater..

[27] Benetti F., Gomes-Filho J.E., de Araujo Lopes J.M., Barbosa J.G., Jacinto R.C., Cintra L.T.A. (2018). In vivo biocompatibility and biomineralization of calcium silicate cements. Eur. J. Oral Sci..

[28] Laghios C.D., Benson B.W., Gutmann J.L., Cutler C.W. (2000). Comparative radiopacity of tetracalcium phosphate and other root-end filling materials. Int. Endod. J..

[29] Xuereb M., Vella P., Damidot D., Sammut C.V., Camilleri J. (2015). In situ assessment of the setting of tricalcium silicate-based sealers using a dentin pressure model. J. Endod..

[30] Cañadas P.S., Berástegui E., Gaton-Hernández P., Silva L.A., Leite G.A., Silva R.S. (2014). Physicochemical properties and interfacial adaptation of root canal sealers. Braz. Dent. J..

[31] Ørstavik D., Nordahl I., Tibballs J.E. (2001). Dimensional change following setting of root canal sealer materials. Dent. Mater..

[32] Caicedo R., von Fraunhofer J.A. (1988). The properties of endodontic sealer cements. J. Endod..

[33] Viapiana R., Flumignan D.L., Guerreiro-Tanomaru J.M., Camilleri J., Tanomaru-Filho M. (2014). Physicochemical and mechanical properties of zirconium oxide and niobium oxide modified Portland cement-based experimental endodontic sealers. Int. Endod. J..

[34] Guerreiro-Tanomaru J.M., Morgental R.D., Flumignan D.L., Gasparini F., Oliveira J.E., Tanomaru-Filho M. (2011). Evaluation of pH, available chlorine content, and antibacterial activity of endodontic irrigants and their combinations against *Enterococcus faecalis*. Oral Sur. Oral Med. Oral Pathol. Oral Radiol. Endod..

[35] Vazquez-Garcia F., Tanomaru-Filho M., Chávez-Andrade G.M., Bosso-Martelo R., Basso-Bernardi M.I., Guerreiro-Tanomaru J.M. (2016). Effect of silver nanoparticles on physicochemical and antibacterial properties of calcium silicate cements. Braz. Dent. J..

[36] Zordan-Bronzel C.L., Tanomaru-Filho M., Rodrigues E.M., Chávez-Andrade G.M., Faria G., Guerreiro-Tanomaru J.M. (2019). Cytocompatibility, bioactive potential and antimicrobial activity of an experimental calcium silicated-based endodontic sealer. Int. Endod. J..

[37] Kim R.J., Shin J.H. (2014). Cytotoxicity of a novel mineral trioxide aggregate-based root canal sealer. Dent. Mater. J..

[38] Martinho F.C., Camargo S.E.A., Fernandes A.M.M., Campos M.S., Prado R.F., Camargo C.H.R., Valera M.C. (2018). Comparison of cytotoxicity, genotoxicity and immunological inflammatory biomarker activity of several endodontic sealers against immortalized human pulp cells. Int. Endod. J..

[39] Silva G.F., Guerreiro-Tanomaru J.M., da Fonseca T.S., Bernardi M.I.B., Sasso-Cerri E., Tanomaru-Filho M., Cerri P.S. (2017). Zirconium oxide and niobium oxide used as radiopacifiers in a calcium silicate-based material stimulate fibroblast proliferation and collagen formation. Int. Endod. J..

[40] Gandolfi M.G., Taddei P., Modena E., Siboni F., Prati C. (2013). Biointeractivity-related versus chemi/physisorption-related apatite precursor-forming ability of current root end filling materials. J. Biomed. Mater. Res. B Appl. Biomater..

[41] Chang S.W., Lee S.Y., Kang S.K., Kum K.Y., Kim E.C. (2014). In vitro biocompatibility, inflammatory response, and osteogenic potential of 4 root canal sealers: Sealapex, Sankin apatite root sealer, MTA Fillapex, and iRoot SP root canal sealer. J. Endod..

[42] Gallo R.L., Dorschner R.A., Takashima S., Klagsbrun M., Eriksson E., Bernfield M. (1997). Endothelial cell surface alkaline phosphatase activity is induced by IL-6 released during wound repair. J. Investig. Dermatol..

